# Direct and indirect relationships between Food Parental Practices, diet quality, and food satisfaction in adolescents

**DOI:** 10.3389/fpubh.2024.1504642

**Published:** 2025-01-30

**Authors:** Carola Del Valle, Horacio Miranda, Ligia Orellana, Cristian Adásme-Berrios, Cristina Calvo-Porral, Berta Schnettler

**Affiliations:** ^1^Doctorado en Ciencias Agroalimentarias y Medioambiente, Facultad de Ciencias Agropecuarias y Medioambiente, Universidad de La Frontera, Temuco, Chile; ^2^Departamento de Producción Agropecuaria, Facultad de Ciencias Agropecuarias y Medioambiente, Universidad de La Frontera, Temuco, Chile; ^3^Centro de Excelencia en Psicología Económica y del Consumo, Universidad de La Frontera, Temuco, Chile; ^4^Departamento de Psicología, Facultad de Educación, Ciencias Sociales y Humanidades, Universidad de La Frontera, Temuco, Chile; ^5^Departamento de Economía y Administración, Universidad Católica del Maule, Talca, Chile; ^6^Business Department, Facultad Economía y Empresa, University of A Coruña, Campus Elviña s/n, Coruña, Spain; ^7^Scientific and Technological Bioresource Nucleus (BIOREN-UFRO), Universidad de La Frontera, Temuco, Chile; ^8^Universidad Católica de Santiago de Guayaquil, Guayaquil, Ecuador

**Keywords:** comprehensive feeding practice questionnaire for adolescents, Healthy Eating Index, satisfaction with food, adolescents, structural mediation model

## Abstract

**Introduction:**

The relationship between four parental feeding practices from the Comprehensive Feeding Practices Questionnaire for adolescents (CFPQ-Teen) and Satisfaction With Food-related Life (SWFoL) in adolescents was evaluated using diet quality measured using the Adapted Healthy Eating Index (AHEI) as a mediating variable.

**Methods:**

Participants were 860 adolescents aged 10–16 years of both sexes who responded to four factors on the CFPQ-Teen, food satisfaction scale, and diet quality index. Structural equation analysis was used in a structural mediation model on a polychoric correlation matrix using the weighted least squares mean-variance adjusted (WLSMV) method.

**Results:**

Diet quality was a mediating factor in the interaction between two parental practices related to parental modeling and adolescent control over SWFoL. There was evidence of a direct relationship between monitoring and restrictive factors for weight control and SWFoL.

**Conclusions:**

The findings indicated that the association between parental feeding practices and food satisfaction may be direct or mediated by diet quality in adolescents.

## 1 Introduction

The population is becoming more aware of the association between healthy eating habits and better health and wellbeing (1). Hence, understanding the factors that influence adolescents' quality of life and wellbeing is increasingly necessary to develop strategies to improve this condition in the early stages of life (2). In the area of food, quality of life has been measured through the Satisfaction With Food-related Life (SWFoL) scale, which assesses people's subjective perception of wellbeing regarding their eating habits (3).

In recent decades, life expectancy has increased considerably, but this has been accompanied by non-communicable diseases such as those associated with overweight and obesity (4). It is a major public health problem for all cultures and age groups (5). Although autonomy in food choices increases during adolescence, adolescents still depend on the food provided by parents and Food Parental Practices (FPP) in which parents exercise at home (6). FPP refer to the specific eating habits parents use to influence what, when, and how much their children eat (7), both during and between meals (8). Healthy eating habits refer to different conditions, such as appropriate times for food intake, hydration, and a varied and healthy diet in portions that correspond to each person according to their age, sex, physical activity, and other contextual variables (9).

A healthy diet is based on one that provides the nutrients that the body needs for its proper functioning, considering the amount of calories, proteins, carbohydrates, lipids, water, and fiber depending on different variables such as age, sex, and stage. Among other conditions, the estimation of a healthy diet can be facilitated by grouping foods based on their nutritional value (e.g., fruits and vegetables, cereals, dairy products, and meat) (10). An unhealthy diet is characterized by an imbalance in the consumption of these nutrients, excessive intake of trans- and saturated fatty meats, refined cereals, high levels of sodium and simple sugars, and deficiency in fruits and vegetables.

In the family context, recent studies have shown that healthy FPP in adolescents can have a positive impact on eating habits (11, 12). FPP, including monitoring and parental modeling, have been associated with improved diet quality in adolescents in various countries (7, 13–17). However, other FPP, such as restriction of weight control, have been associated with unhealthy diets and higher weight (43). Other studies have reported a negative association between adolescent control and diet quality, because parents control less of what adolescents eat in response to their quest for autonomy in food choice (11), leading to a decrease in adolescents' diet quality (44).

Variables that are positively related to higher SWFoL include healthy eating habits and better diet quality (7, 13, 16–19). Similarly, several studies have suggested a positive association between the FPP that parents apply for home and SWFoL (20, 21). In addition, Vaughn et al. (22) suggested that there is evidence linking structural FPP with non-obesogenic environments, whereas McGowan et al. (23) asserted that the monitoring factor acts as a predictor of fruit consumption.

The background presented indicates that diet quality is associated with FPP and SWFoL in adolescents, and that FPP are also associated with SWFoL. However, the relationships between FPP, diet quality, and SWFoL have not been explored.

The CFPQ-Teen was used, which Piccoli et al. (6) adapted to Portuguese, to measure the perception of FPP in Brazilian adolescents. This study used four latent factors—monitoring, parental modeling, weight control restriction, and adolescent control—adapted and validated by Del Valle et al. (43) to be answered by teenagers in Chile.

Based on the above, this study aimed to assess the direct and indirect relationships between the four FPP on the CFPQ-Teen and SWFoL mediated by the AHEI in adolescents in two cities in Chile.

Based on the study objectives, the following hypotheses were established:

- H1: Monitoring FPP are directly and positively associated with SWFoL in adolescents.
- H2: Monitoring FPP are directly and positively associated with AHEI in adolescents.
- H3: Adolescent control of FPP are directly and negatively associated with SWFoL.
- H4: Adolescent control of FPP are directly and negatively associated with AHEI.
- H5: Parental modeling of FPP are directly and positively associated with SWFoL in adolescents.
- H6: Parental modeling of FPP are directly and positively associated with AHEI in adolescents.
- H7: Restriction of weight control of FPP are directly and negatively associated with SWFoL in adolescents.
- H8: Restriction of weight control of FPP are directly and negatively associated with diet quality in adolescents.
- H9: The AHEI has an indirect relationship with the CFPQ-Teen FPP and SWFoL in adolescents.

## 2 Methods

### 2.1 Sampling and procedure

The participants were selected through proportional quota sampling in order to access a sample that reflected the communal distribution of families according to socioeconomic level (high, medium, and low) in Temuco and Santiago, Chile. Prior to data collection, parents were asked to sign a consent form authorizing the participation of a child. The children were asked to sign the consent form. Consent and assent will ensure voluntary participation and endorse the confidentiality and anonymity of the data obtained. The inclusion criteria for this study stipulated that the participants were adolescents aged 10–16 years who came from households with one father and one mother, each of whom contributed to household income. Participants were recruited through contact with the authorities of educational establishments located in urban areas between March and July 2021 in Santiago (*n* = 430) and between July and December 2021 in Temuco (*n* = 430), Chile. The questionnaires were hosted on the QuestionPro platform (QuestionPro Inc.) and were sent by email to the adolescents' mothers by previously trained interviewers. After receiving the completed questionnaire, each family received USD 15. The Ethics Committee of the University of La Frontera approved the study protocol (Protocol Number 007/2019).

### 2.2 Instruments

In this study, a four-factor model of the CFPQ-Teen was used, adapted, and validated by Del Valle et al. (43) to be answered by adolescents. This model is composed of 20 items representing four factors of the CFPQ-Teen, which are represented by four items that assess adolescents' perception of the frequency with which parents monitor the consumption of unhealthy foods; the adolescent control factor is represented by four items that assess the frequency with which parents are more permissive concerning adolescent eating behavior and habits; the weight control restriction factor is represented by eight items that assess adolescents' perception of control over food intake to reduce or maintain their weight; and the parental modeling factor is represented by four items that determine how adolescents perceive their parents as a model or reference for them in terms of healthy eating habits. Del Valle et al. (43) obtained the following McDonald's omega for each factor of the Spanish-validated model: Monitoring = 0.91, Adolescent Control = 0.69, Restriction for Weight Control = 0.90, and Parental Modeling = 0.83.

Adolescent control factor items (e.g., “Do your parents allow you to eat whatever you want?”) and Monitoring [e.g., “How often do your parents check the number of sweets (or ice cream, cakes, chocolates, candies, pies, pastries) you eat?”] were answered on a 5-point Likert-type scale, from 1 “never” to 5 “always.” The factors Restrictions for weight control (e.g., “Do my parents need to be sure I don't eat high-fat foods?”) and parental modeling (e.g., “My parents eat healthy food to give me an example of healthy eating.”) were answered on a 5-point Likert-type scale, from 1 “strongly disagree” to 5 “strongly agree” (Supplementary Table 1).

The Adapted Healthy Eating Index (AHEI) is an adaptation of the US-HEI (10) developed by Norte and Ortiz (24) for the qualitative estimation of diet quality in Spanish-speaking populations. Participants were asked to respond to the AHEI variables indicating the frequency of consumption of the nine food groups: (1) Cereals and by-products, (2) Vegetables, (3) Fruits, (4) Milk and dairy products, (5) Meats, (6) Legumes, (7) Cold cuts and sausages, (8) Sweets, (9) Sugary drinks, and (10) Varieties of diets. The consumption frequency data for each food group were converted into a score from 0 to 10, according to the degree of compliance with the dietary recommendations. The AHEI score was calculated by adding the scores for each variable. The AHEI variable scores add up to a maximum of 100 points. Scores over 80 are indicative of a “healthy” diet; scores between 51 and 80 correspond to a diet that “requires change”; scores below 50 correspond to “unhealthy” diets (10) (Supplementary Table 2).

Satisfaction With Food-related Life (SWFoL) (3) comprises five items grouped into a single dimension that assesses a person's subjective perception of wellbeing concerning food and eating habits. The validated Spanish version of the SWFoL used in this study (25) showed good internal consistency in samples of adolescents in Chile (Cronbach's α = 0.89–0.91) (20, 26–28). Respondents indicated their degree of agreement with each statement on a 6-point Likert scale (e.g., food and meals offer great satisfaction in your daily life) from 1 “completely disagree” to 6 “completely agree” (Supplementary Table 3).

### 2.3 Data analysis

This study used a cross-sectional, nonexperimental, descriptive, correlational, and functional dependency design. Statistical Package for the Social Sciences (IBM SPSS) v. 23 was used for descriptive analyses.

The Mplus v. 8.4. software was used to analyze the structural mediation model. The weighted least squares mean-variance adjusted (WLSMV) method was used to estimate the parameters of this model, considering the ordinal response scale of the items and the optimization of the standard errors of the PATH (8, 29–32, 42).

The mediation effect was evaluated through structural equation modeling using AHEI as the mediating variable between the four factors on the CFPQ-Teen and SWFoL. The statistical significance of indirect associations in the model was verified through confidence intervals using the bootstrap method with an estimation of 5,000 iterations (33). The global and incremental goodness-of-fit indicators of the structural model, RMSEA, CFI, and TLI, were estimated using WLSMV to analyze non-normally distributed data (34).

## 3 Results

### 3.1 Descriptive analysis

The sample of 860 adolescents in Santiago and Temuco comprised 434 male and 426 female adolescents was composed of 50.5% male adolescents and 49.5% female adolescents, with a mean age of the adolescents of 13.04 (SD = 2.05) and adolescents 13.28 (SD = 2.02). The families had a mean number of family group members of 4.38 (SD = 1.36), and families with a medium socioeconomic level (81.3%). The SWFoL variable had a mean of 23.37 (SD = 4.77), the CFPQ-Teen factors presented means of Monitoring of 13.79 (SD = 4.75), adolescent control of 10.55 (SD = 3.43), parental model of 15.07 (SD = 3.79), and weight control restriction of 22.18 (SD = 8.06). The AHEI presented a mean of 63.58 (SD = 13.97). The AHEI measurement in adolescents showed that 10.58% of the participants of both sexes presented a “healthy” diet, while 72.56% “require changes” in their diet, and 16.86% had an “unhealthy diet” (Table 1).

**Table 1:** Sample characteristics: centralization and dispersion values.

**Variable analyzed (*n* = 860)**	**Value**
**Adolescent age [mean (SD)]**	
Male	13.04 (2.05)
Female	13.28 (2.02)
**Adolescent sex (%)**	
Male	50.5
Female	49.5
Family members [mean (SD)]	4.38 (1.36)
Number of children [mean (SD)]	2.18 (0.87)
**Socioeconomic status (%)**	
High	5.0
Medium	81.3
Low	13.7
SWFoL [mean (SD)]	23.37 (4.77)
Monitoring [mean (SD)]	13.79 (4.75)
Adolescent control [mean (SD)]	10.55 (3.43)
Parental modeling [mean (SD)]	15.07 (3.79)
Restriction for weight control [mean (SD)]	22.18 (8.06)
AHEI [mean (SD)]	63.58 (13.97)

### 3.2 Relationships between FPP, diet quality, and food satisfaction

This study's structural mediation model presented medium to high goodness-of-fit levels: CFI: 0.914, TLI: 0.902, and RMSEA: 0.065. Convergent validity demonstrated that all saturations were statistically significant (*p* ≤ 0.05).

The four independent factors of the CFPQ-Teen questionnaire with the description of all indicator variable names (mo1 to wr39) and their relations with theory are found in Table 2.

**Table 2:** Factors, prefix, variables and description for CFPQ-Teen (43).

**Factores**	**Items**
Monitoring	**(mo1)** How often does this caregiver keep track of the quantity of sweets (or ice cream, cakes, pies, chocolates, candies) that you eat?
	**(mo2)** How often does this caregiver keep track of the quantity of industrialized snacks (potato chips, munchies, cheese pastries, etc.) that you eat?
	**(mo3)** How often does this caregiver keep track of the quantity of fatty foods (hamburgers, snacks, mayonnaise, etc.) that you eat?
	**(mo4)** How often does s/he keep track of the quantity of sweet drinks (soda/soft drinks, juices) that you drink?
Adolescent control	**(ac6)** This caregiver allows you to eat whatever you want?
	**(ac10)** Can you choose the items you want of what is served at lunch or dinner, leaving aside what you do not like, without interference from the caregiver?
	**(ac11)** When you do not like what is served for eating, does your caregiver cook something else for you?
	**(ac12)** Does this caregiver allow you to have snacks whenever you want?
Restriction for weight control	**(rw18)** This caregiver needs to be sure that I do not eat fatty foods
	**(rw24)** This person encourages me to eat less food so that I won't get fat
	**(rw26)** This person helps me control the quantity of food that I serve myself at each meal in order to control my weight
	**(rw29)** If I eat more than normal at one meal, this person reduces the quantity of food at the next meal
	**(rw30)** This caregiver limits the foods that might make me fat
	**(rw31)** S/he believes that I should not eat certain foods so that I do not gain weight
	**(rw36)** I am monitored so that I do not eat between meals in order to not get fat
	**(rw39)** This caregiver forces me to restrict my diet in order to control my weight
Parental modeling	**(pm38)** This caregiver eats healthy food to give me an example of healthy eating habits
	**(pm40)** Even when it is not the caregiver's preferred food, s/he often eats it because s/he finds it important to give me her/his example
	**(pm41)** S/he tries to show enthusiasm regarding healthy food
	**(pm42)** S/he shows me how much s/he enjoys eating healthy food

Prefix: Monitoring = mo01, 02, 03, 04; Adolescent Control = ac05, 06, 10, 11; Restriction for Weight Control = wr18, 24, 26, 29, 30, 31, 36, 39; Parental Modeling = pm38, 40, 41, 42.

Adolescent control factors and Monitoring factors were answered on a 5-point Likert-type scale, from 1 “never” to 5 “always.” The factors Restriction for weight control and parental modeling factors were answered on a 5-point Likert-type scale, from 1 “strongly disagree” to 5 “strongly agree”.

**Figure 1:**
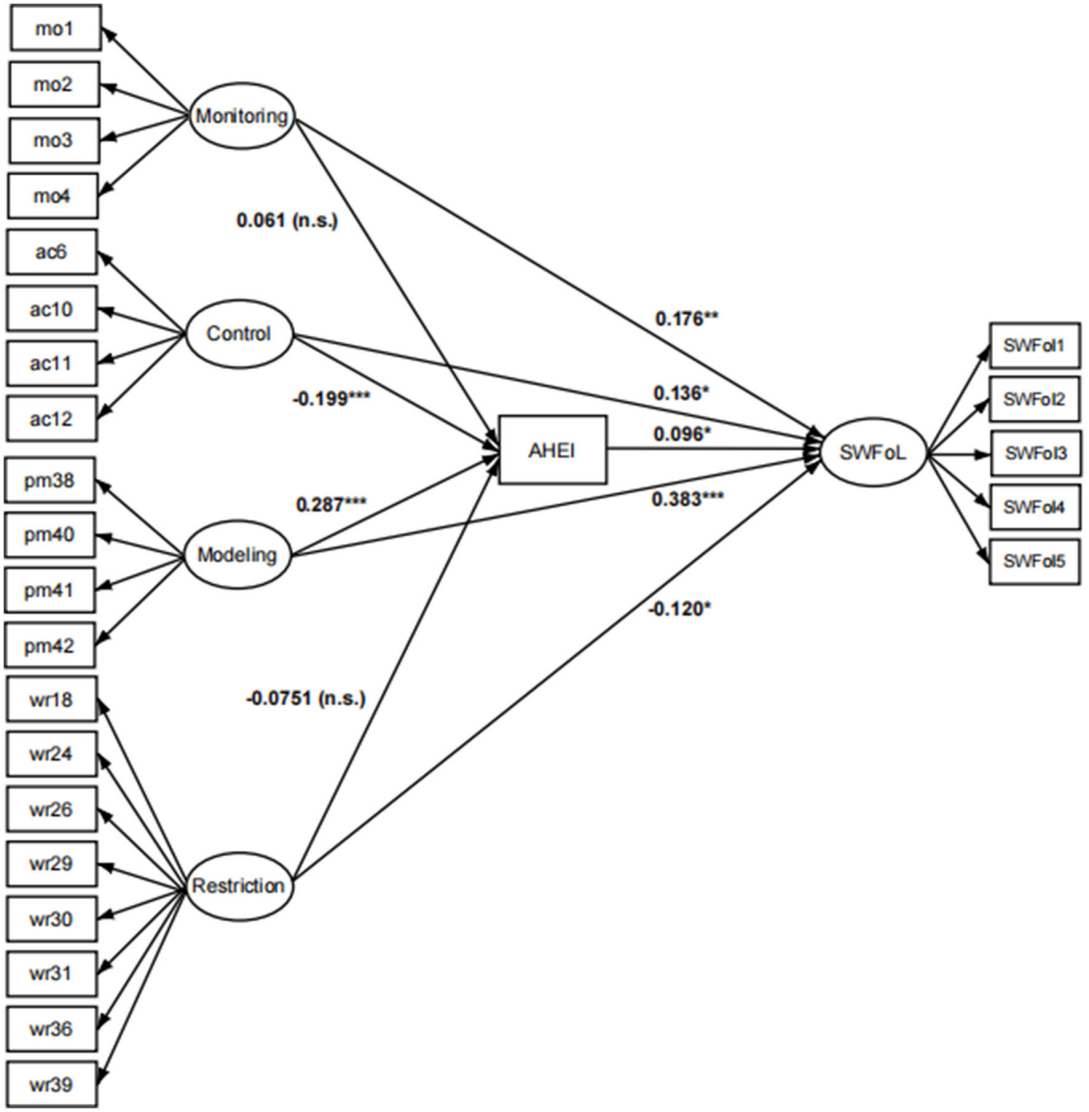
Standardized structural diagram of the model relating four parental feeding practices from the CFPQ-Teen, feeding quality (measured with the AHEI), and Satisfaction with food-related life (SWFoL) in adolescents. Monitoring = mo01, 02, 03, 04; Adolescent Control = ac05, 06, 10, 11; Parental Modeling = pm38, 40, 41, 42; Restriction for weight control = wr18, 24, 26, 29, 30, 31, 36, 39. Statistical significance: * *p*-value ≤ 0.05, ** *p*-value ≤ 0.01, *** *p*-value ≤ 0.001.

The PATH values and statistical significances of the direct and indirect relationships between the CFPQ-Teen factors with SWFoL and AHEI are shown in Figure 1.

The monitoring factor showed a positive and highly significant direct relationship with SWFoL 0.068 (*p* ≤ 0.01), thus confirming H1, which establishes a positive relationship between monitoring and SWFoL in adolescents, and showed a positive and non-significant relationship with diet quality, 0.294 (*p* > 0.05), thus rejecting H2. The indirect effect of SWFoL through AHEI was positive and insignificant, as it included zero at 95% CI 0.002 (−0.001; 0.008), which ruled out H9; therefore there would be no indirect relationship of monitoring on SWFoL mediated by the AHEI.

The adolescent control factor showed a positive and highly significant direct relationship with SWFoL 0.097 (*p* ≤ 0.01); this confirms the significance but not the direction of the relationship in H3, which was negative, so that the control factor would positively influence food satisfaction in adolescents. Also the factor adolescent control showed a negative and very highly significant relationship with the AHEI, −1.738 (*p* ≤ 0.001), which confirms H4 that establishes that there is a negative and significant relationship between monitoring and the AHEI. The indirect relationship between adolescent control and SWFoL through the AHEI was negative and significant, as it did not include zero, with a 95% CI of −0.014 (−0.030; −0.001), confirming H9 that there would be an indirect relationship between adolescent control and SWFoL mediated by the AHEI.

The parental modeling factor showed a positive and highly significant direct relationship with SWFoL 0.346 (*p* ≤ 0.001), thus confirming H5, which establishes a positive relationship between the parental modeling factor and SWFoL in adolescents, as well as a positive and highly significant relationship with el AHEI 3.193 (*p* ≤ 0.001), confirming H6, which establishes that there is a positive and significant relationship between parental modeling and AHEI. The indirect relationship between the parental modeling factor and SWFoL through the AHEI was positive and significant, as it did not include zero with a 95% CI of 0.025 (0.003; 0.049), confirming H9 that there would be an indirect parental modeling factor relationship on SWFoL mediated by the AHEI.

The restriction for weight control factor showed a negative and significant direct relationship with SWFoL −0.164 (*p* ≤ 0.05), thus confirming H7, which establishes a negative and significant relationship between restriction for weight control factor and SWFoL in adolescents; also, the restriction for weight control factor showed a negative and non-significant relationship with the AHEI, −1.259 (*p* > 0.05), thus rejecting H8. The indirect relationship with SWFoL through AHEI was negative and insignificant, as it included zero at a 95% CI of −0.010 (−0.027; 0.001), thus rejecting H9. Therefore, there would be no indirect relationship between the restriction of weight control factors and SWFoL mediated by the AHEI (Table 3).

**Table 3:** Unstandardized estimates of the factors of the structural mediation model.

**Factor**	**Total effect**	**Direct effect**	**PATH a**	**PATH b**	**Indirect effect**	**LCI 0.95**	**UCI 0.95**
Monitoring^a^	0.071^**^	0.068^**^	0.294	0.008^*^	0.002	−0.001	0.008
Monitoring^b^	0.182	0.176	0.061	0.096	0.006	−0.006	0.017
Control^a^	0.083^***^	0.097^**^	−1.738^***^	0.008^*^	−0.014	−0.030	−0.001
Control^b^	0.117	0.136	−0.199	0.096	−0.019	−0.038	−0.001
Modeling^a^	0.371^***^	0.346^***^	3.193^***^	0.008^*^	0.025	0.003	0.049
Modeling^b^	0.410	0.383	0.287	0.096	0.027	0.003	0.052
Restriction^a^	−0.174^*^	−0.164^*^	−1.259	0.008^*^	−0.010	−0.027	0.001
Restriction^b^	−0.127	−0.120	−0.075	0.096	−0.007	−0.018	0.003

^a^Unstandardized value.

^b^Standardized value.

Total effect, simple regression coefficient without mediation or covariates; Direct effect, coefficient of simple regression with mediation and covariates; PATH a, coefficient of simple regression on the mediating variable; PATH b, coefficient of simple regression of the mediating variable on the dependent variable; Indirect effect, product of a × b; LCI, lower confidence interval; UCI, upper confidence interval.

Statistical significance: ^*^p-value ≤ 0.05, ^**^p-value ≤ 0.01, ^***^p-value ≤ 0.001.

The relationships between the factors and the PATH estimated for each hypothesis are shown in Table 4.

**Table 4:** Relationships between factors, PATH for each hypothesis test.

**Relationships**	**Variables**					**PATH**		**Hypothesis test**
	**Independent**	**Mediating**			**Dependent**	**Standardized**	**Unstandardized**	
Direct	Monitoring		→		SWFoL	0.176	0.068^**^	H1: Confirming
Direct	Monitoring		→		AHEI	0.061	0.294	H2: Rejecting
Indirect	Monitoring	→	AHEI	→	SWFoL	0.006	0.002	H9: Rejecting
Direct	Control		→		SWFoL	0,136	0.097^**^	H3: Confirming
Direct	Control		→		AHEI	−0.199	−1.738^***^	H4: Confirming
Indirect	Control	→	AHEI	→	SWFoL	−0.019	−0.014^*^	H9: Confirming
Direct	Modeling		→		SWFoL	0.383	0.346^***^	H5: Confirming
Direct	Modeling		→		AHEI	0.287	3.193^***^	H6: Confirming
Indirect	Modeling	→	AHEI	→	SWFoL	0.027	0.025	H9: Confirming
Direct	Restriction		→		SWFoL	−0.120	−0.164^*^	H7: Confirming
Direct	Restriction		→		AHEI	−0.075	−1.259	H8: Rejecting
Indirect	Restriction	→	AHEI	→	SWFoL	−0.007	−0.010	H9: Rejecting

Statistical significance: ^*^p-value ≤ 0.05, ^**^p-value ≤ 0.01, ^***^p-value ≤ 0.001, (n.s.), p-value > 0.05 (R#4).

## 4 Discussion and conclusion

This study aimed to assess the direct and indirect relationships between four FPP on the CFPQ-Teen and SWFoL mediated by the AHEI in adolescents in two cities in Chile. When this study was conducted, to the best of the authors' knowledge, no study had been reported that used a structural equation model to analyze the relationship of diet quality mediated through the AHEI between four FPP on the CFPQ-Teen and the SWFoL.

Regarding H1, which states that FPP Monitoring is positively associated with SWFoL in adolescents, the results observed in the sample presented a positive and statistically significant relationship, which allows us to confirm H1, concluding that the monitoring factor improves SWFoL in adolescents.

In relation to H2, which states that Monitoring FPP are positively associated with AHEI in adolescents, the results observed in the sample present a non-significant relationship, which allowed H2 to be rejected, concluding that the monitoring factor does not present an indirect relationship with SWFoL through AHEI. This result contrasts with those of studies that associate FPP with better diet quality (7, 16). This finding may be due to adolescents at this stage of life exhibiting maladaptive eating behaviors (35) and disordered eating habits (36), leading to a decrease in diet quality (37–39). Maladaptive eating behaviors in adolescence can be partially attributed to the increased autonomy and independence attained during this developmental stage, including dietary choices (6, 11).

In relation to H3, which states that Adolescent control FPP are negatively associated with SWFoL in adolescents, the results observed in the sample presented a direct positive relationship, which is in contrast with the direction of H3, concluding that the adolescent control factor improves SWFoL.

In relation to H4, which states that Adolescent control FPP is negatively associated with AHEI in adolescents, the results observed in the sample allowed H4 to be preserved, evidencing a direct and negative relationship with the AHEI, concluding that the relationship of the adolescent control factor on SWFoL presents a suppression mediation relationship through the AHEI. The indirect relationship between the adolescent control factor and SWFoL via the AHEI corresponds to a suppression-type relationship, given that the direct relationship was greater than the total relationship, and there were also relationships with opposite signs with the AHEI and between the AHEI and SWFoL (40).

In relation to H5, which states that Parental modeling of FPP are positively associated with SWFoL in adolescents, the results observed in the sample presented a positive and statistically significant relationship, which allows us to confirm H5, concluding that the parental modeling factor improves SWFoL in adolescents. These results are consistent with studies that have shown that FPP, such as parental modeling for adolescents, can have a positive impact on diet quality (11, 12, 14).

In relation to H6, which states that Parental modeling FPP are positively associated with AHEI in adolescents, the results observed in the sample presented a positive and statistically significant relationship, which allows us to confirm H6, concluding that the parental modeling factor is related to the SWFoL indirectly and partially through AHEI in adolescents.

In relation to H7, which states that Restriction for weight control FPP are negatively associated with SWFoL in adolescents, the results observed in the sample presented a negative and statistically significant relationship, which allowed us to confirm H7, concluding that factor restriction for weight control reduces SWFoL in adolescents.

In relation to H8, which states that the Restriction for weight control FPP is negatively associated with AHEI in adolescents, the results observed in the sample present a non-significant relationship, which allowed H8 to be rejected, concluding that the factor restriction for Weight control does not present an indirect relationship with SWFoL through AHEI.

In this study, there were no statistically significant differences in the indirect relationship between weight control restriction and SWFoL mediated by the AHEI. This aligns with other research indicating that adolescents tend to reject coercive FPP, which is linked to increased body weight (7, 41).

In relation to H9, which states that AHEI has an indirect relationship between CFPQ-Teen FPP and SWFoL in adolescents, the results observed in the sample presented indirect and significant relationships; the AHEI fulfills a mediating role only through adolescent control and parental modeling factors on the CFPQ-Teen with SWFoL.

Based on the results, it can be concluded that the four factors of the CFPQ-Teen have a direct relationship with the SWFoL. There was evidence of an indirect relationship between adolescent control and parental modeling and SWFoL through the AHEI. Consequently, while all four FPP evaluated are associated with adolescents' subjective wellbeing in the dietary domain, only two exert an indirect influence through AHEI. Adolescent control has a negative association with AHEI because parents control what adolescents eat less in response to their search for autonomy in food choice (11), reducing the quality of their adolescents' diet (44). The parental modeling factor was associated with better diet quality in adolescents, which is consistent with previous studies with samples of adolescents from different countries (7, 13–17, 41).

A remarkable aspect of this study is that it was conducted with a large sample size (*n* = 860), which resulted in greater accuracy in estimating the parameters and global and incremental psychometric goodness-of-fit indices. This study used a polychoric correlation matrix for categorical variables with an ordinal Likert-type response by applying the WLSMV method to optimize the standard error of estimating saturations in structural equation model analyses.

The limitations of this study include the fact that the AHEI does not measure the amount of food consumed or energy value, nor does it consider contextual variables, such as ethnicity, parental educational level, age, etc. The AHEI measures the frequency of consumption by food groups, which is considered an indicator of diet quality and nutritional health (24). Also provides evidence on the food consumption pattern in the country that applies (10). In Chile it is used by the Ministry of Health to measure the quality of the population's diet and has been used in numerous studies in adolescents (14, 15).

This study contributes to the application of research on eating behavior, generating a contribution to public health, specifically at the level of the Ministry of Health, health institutions, and educational institutions, to generate educational strategies for the application of healthy eating habits. Different interventions, considering that the AHEI provides evidence of the type of foods consumed, allow the construction of dietary guides to direct nutritional education in a more comprehensive way through nutritional assistance programs applied to adolescents and their parents to reduce obesity in adolescents in Chile. The study of perceptions about contributes to the construction of scenarios that include the mediating effect of the AHEI and its implications on SWFoL, considering eating habits in the orientation of the food industry in governmental and non-governmental organizations. These results constitute a relevant contribution to advance future research that promotes healthy eating habits in families with adolescent children in Latin America.

This shows the importance of including diet quality as a mediating variable in the relationship between CFPQ-Teen factors, parental model, weight control restriction, and SWFoL. This impacts structural models in future studies by necessitating the inclusion of the AHEI in the examination of FPP and SWFoL among adolescents.

## Data Availability

The raw data supporting the conclusions of this article will be made available by the authors, without undue reservation.

## References

[1] de Albuquerque Araújo L Álvarez HAJ Palomo GI Bustamante UMA. Determinantes de la satisfacción con la alimentación en adultos mayores chilenos [Determinants of satisfaction with food-related life in older Chileans adults]. Nutr Hosp. (2019) 36:805–12. 10.20960/nh.02481 31232574

[2] Liu R Grunert KG. Satisfaction with food-related life and beliefs about food health, safety, freshness and taste among the elderly in China: a segmentation analysis. Food Qual Prefer. (2020) 79:103775. 10.1016/j.foodqual.2019.103775

[3] Grunert KG Dean M Raats MM Nielsen NA Lumbers M. A measure of satisfaction with food-related life. Appetite. (2007) 49:486–93. 10.1016/j.appet.2007.03.010 17481776

[4] Fernandes DPS Lopes Duarte MS Pessoa MC Castro Franceschini SC Queiroz Ribeiro A. Healthy Eating Index: assessment of the diet quality of a Brazilian elderly population. Nutr Metab Insights. (2018) 11:117863881881884. 10.1177/1178638818818845 30626998 PMC6311538

[5] Adasme-Berrios C Carreño C Aliaga-Ortega L Schnettler B Lobos G. Factores que determinan la elección de alimentos procesados por estudiantes universitarios en el contexto de las etiquetas de advertencia nutricional. Rev Chil Nutr. (2022) 49:451–8. 10.4067/S0717-7518202200050045127315006

[6] Piccoli ÂB Neiva-Silva L Mosmann CP Musher-Eizenman D Pellanda LC. Adolescents' perception of parental feeding practices: adaptation and validation of the comprehensive feeding practices questionnaire for Brazilian adolescents-The CFPQ-Teen. PLoS ONE. (2017) 12:e0187041. 10.1371/journal.pone.0187041 29145485 PMC5690605

[7] Costarelli V Michou M Panagiotakos DB Lionis C. Adherence to the Mediterranean diet and weight status in children: the role of parental feeding practices. Int J Food Sci Nutr. (2021) 72:112–22. 10.1080/09637486.2020.1765151 32458711

[8] Yang WY Burrows T MacDonald-Wicks L Williams LT Collins CE Chee WSS. Parent-child feeding practices in a developing country: findings from the Family Diet Study. Appetite. (2018) 125:90–7. 10.1016/j.appet.2018.01.037 29408380

[9] Abdel-Megeid FY Abdelkarem HM El-Fetouh AM. Unhealthy nutritional habits in university students are a risk factor for cardiovascular diseases. Saudi Med J. (2011) 32:621–7. 21666946

[10] Kennedy ET Ohls J Carlson S Fleming K. The Healthy Eating Index: design and applications. J Am Diet Assoc. (1995) 95:1103–8. 10.1016/S0002-8223(95)00300-2 7560680

[11] Fleary SA Ettienne R. The relationship between food parenting practices, parental diet and their adolescents' diet. Appetite. (2019) 135:79–85. 10.1016/j.appet.2019.01.008 30639293

[12] Yee AZH Lwin MO Ho SS. Promoting healthier eating via parental communication: development and validation of the active and restrictive Parental Guidance Questionnaire (PARQ). Health Commun. (2021) 36:1514–26. 10.1080/10410236.2020.1773696 32530309

[13] Melbye EL Hansen H. Child weight and parental feeding practices: a child-responsive model. Nutr Food Sci. (2015) 45:174–88. 10.1108/NFS-08-2014-0074

[14] Schnettler B Miranda-Zapata E Orellana L Saracostti M Poblete H Lobos G. Parents' modeling during the COVID-19 pandemic: influences on family members' diet quality and satisfaction with-food-related life in dual-earner parents with adolescent children. Front Nutr. (2022) 9:902103. 10.3389/fnut.2022.902103 35662953 PMC9158745

[15] Schnettler B Orellana L Miranda-Zapata E Saracostti M Poblete H Lobos G. Contributions of work-to-family enrichment to parental food monitoring and satisfaction with food-related life during the COVID-19 pandemic in dual-earner parents and their adolescent children. Nutrients. (2022) 14:4140. 10.3390/nu14194140 36235792 PMC9572603

[16] Thomson JL Hennessy E Landry AS Goodman MH. Patterns of food parenting practices regarding junk food and sugary drinks among parent-child dyads. Nutr J. (2020) 19:91. 10.1186/s12937-020-00610-3 32847599 PMC7448982

[17] Wrobleski MM Parker EA Hurley KM Oberlander S Merry BC Black MM. Comparison of the HEI and HEI-2010 diet quality measures in association with chronic disease risk among low-income, African American urban youth in Baltimore, Maryland. J Am Coll Nutr. (2018) 37:201–8. 10.1080/07315724.2017.1376297 29313747 PMC6167057

[18] Cabezas MF Nazar G. Asociación entre autorregulación alimentaria, dieta, estado nutricional y bienestar subjetivo en adultos en Chile. Ter Psicol. (2022) 40:1–21. 10.4067/s0718-4808202200010000127315006

[19] Vilugrón Aravena F Molina GT Gras Pérez ME Font-Mayolas S. Hábitos alimentarios, obesidad y calidad de vida relacionada con la salud en adolescentes chilenos. Rev Med Chile. (2020) 148:921–9. 10.4067/S0034-98872020000700921 33399676

[20] Schnettler B Lobos G Miranda-Zapata E Denegri M Ares G Hueche C. Diet quality and satisfaction with life, family life, and food-related life across families: a cross-sectional pilot study with mother-father-adolescent triads. Int J Environ Res Public Health. (2017) 14:1313. 10.3390/ijerph14111313 29109387 PMC5707952

[21] Schnettler B Miranda-Zapata E Orellana L Poblete H Lobos G Lapo M. Domain satisfaction and overall life satisfaction: testing the spillover-crossover model in Chilean dual-earner couples. Int J Environ Res public Health. (2020) 17:7554. 10.3390/ijerph17207554 33080810 PMC7589047

[22] Vaughn AE Ward DS Fisher JO Faith MS Hughes SO Kremers SPJ. Fundamental constructs in food parenting practices: a content map to guide future research. Nutr Rev. (2016) 74:98–117. 10.1093/nutrit/nuv061 26724487 PMC4892304

[23] McGowan L Croker H Wardle J Cooke LJ. Environmental and individual determinants of core and non-core food and drink intake in preschool-aged children in the United Kingdom. Eur J Clin Nutr. (2012) 66:322–8. 10.1038/ejcn.2011.224 22252108 PMC3378504

[24] Norte Navarro AI Ortiz Moncada R. [Spanish diet quality according to the healthy eating index]. Nutr Hosp. (2011) 26:330–6. 10.1590/S0212-16112011000200014 21666971

[25] Schnettler B Miranda H Sepúlveda J Denegri M Mora M Lobos G. Psychometric properties of the satisfaction with food-related life scale: application in southern Chile. J Nutr Educ Behav. (2013) 45:443–9. 10.1016/j.jneb.2012.08.003 23337474

[26] Schnettler B Grunert KG Lobos G Miranda-Zapata E Denegri M Hueche C. Maternal food-related practices, quality of diet, and well-being: profiles of chilean mother-adolescent dyads. J Nutr Educ Behav. (2018) 50:776–87. 10.1016/j.jneb.2018.03.003 29625914

[27] Schnettler B Miranda-Zapata E Lobos G Saracostti M Denegri M Lapo M. The mediating role of family and food-related life satisfaction in the relationships between family support, parent work-life balance and adolescent life satisfaction in dual-earner families. Int J Environ Res Public Health. (2018) 15:2549. 10.3390/ijerph15112549 30441763 PMC6266111

[28] Schnettler B Miranda H Miranda-Zapata E Lobos G Denegri M Lapo M. Diet quality and satisfaction with different domains of life in single- and dual-headed households: comparing mother-adolescent dyads. Child Youth Serv Rev. (2018) 89:124–31. 10.1016/j.childyouth.2018.04.027

[29] Li C-H. The performance of ML, DWLS, and ULS estimation with robust corrections in structural equation models with ordinal variables. Psychol Methods. (2016) 21:369–87. 10.1037/met0000093 27571021

[30] Li CH. Confirmatory factor analysis with ordinal data: comparing robust maximum likelihood and diagonally weighted least squares. Behav Res Methods. (2016) 48:936–49. 10.3758/s13428-015-0619-7 26174714

[31] Rhemtulla M Brosseau-Liard PÉ Savalei V. When can categorical variables be treated as continuous? A comparison of robust continuous and categorical SEM estimation methods under suboptimal conditions. Psychol Methods. (2012) 17:354–73. 10.1037/a0029315 22799625

[32] Yang-Wallentin F Joreskog K Luo H. Confirmatory factor analysis of ordinal variables with misspecified models. Struct Equ Modeling. (2010) 17:392–423. 10.1080/10705511.2010.489003

[33] Efron B. Bootstrap methods: another look at the jackknife. Ann Stat. (1979) 7. 10.1214/aos/117634455238281721

[34] Li C-H. Statistical estimation of structural equation models with a mixture of continuous and categorical observed variables. Behav Res Methods. (2021) 53:2191–213. 10.3758/s13428-021-01547-z 33791955

[35] Powell EM Frankel LA Hernandez DC. The mediating role of child self-regulation of eating in the relationship between parental use of food as a reward and child emotional overeating. Appetite. (2017) 113:78–83. 10.1016/j.appet.2017.02.017 28215543

[36] Lim CS Anderson LM Hollingsworth DW Shepherd L Sandridge S Lanciers S. Comparing disordered eating and feeding practices in African American and Caucasian treatment-seeking youth with obesity. Eat Disord. (2019) 27:152–67. 10.1080/10640266.2019.1614825 31084424 PMC6815514

[37] Aliaga-Ortega L Adasme-Berríos C Méndez C Soto C Schnettler B. Processed food choice based on the theory of planned behavior in the context of nutritional warning labels. Br Food J. (2019) 121:3266–80. 10.1108/BFJ-10-2018-0695

[38] Chansukree P Rungjindarat N. Social cognitive determinants of healthy eating behaviors in late adolescents: a gender perspective. J Nutr Educ Behav. (2017) 49:204–10.e1. 10.1016/j.jneb.2016.10.019 28284358

[39] Quattlebaum M Wilson DK Sweeney AM Zarrett N. Moderating effects of parental feeding practices and emotional eating on dietary intake among overweight african american adolescents. Nutrients. (2021) 13:1920. 10.3390/nu13061920 34204927 PMC8229013

[40] MacKinnon DP Krull JL Lockwood CM. Equivalence of the mediation, confounding and suppression effect. Prev Sci. (2000) 1:173–81. 10.1023/a:1026595011371 11523746 PMC2819361

[41] Haszard JJ Williams SM Dawson AM Skidmore PM Taylor RW. Factor analysis of the comprehensive feeding practices questionnaire in a large sample of children. Appetite. (2013) 62:110–8. 10.1016/j.appet.2012.11.017 23207187

[42] Yoo B. Cross-group comparisons: a cautionary note. Psychol Mark. (2002) 19:357–68. 10.1002/mar.10014

[43] Del Valle C Miranda H Orellana L Grunet KG Adasme-Berrios C Schnettler B. Children's perception of food parenting practices: adaptation and validation of the comprehensive feeding practices questionnaire in Chilean adolescents. Front Public Health. (2024) 12:1343623. 10.3389/fpubh.2024.1343623 38544728 PMC10972623

[44] Gunther C Reicks M Banna J Suzuki A Topham G Richards R. Food parenting practices that influence early adolescents' food choices during independent eating occasions. J Nutr Educ Behav. (2019) 51:993–1002. 10.1016/j.jneb.2019.05.597 31221526

