# Supplementary material for: Direct and indirect relationships between Food Parental Practices, diet quality, and food satisfaction in adolescents
Source: Front Public Health. 2025 Jan 30;12:1504642. doi: 10.3389/fpubh.2024.1504642 (PMC11822477; doi:10.3389/fpubh.2024.1504642)
Supplement: Supplementary file 1 [file Table_1.doc]

Supplementary Material

# Supplementary Tables

**CFPQ-Teen (Del Valle et al., 2024)**

| **Factores** | **Ítems** |
| --- | --- |
| **Monitoreo** | How often does this caregiver keep track of the quantity os sweets (or ice cream, cakes, pies, chocolates, candies) that you eat? (1) |
|  | ¿Con que frecuencia tus padres revisan la cantidad de dulces (o helados, pasteles, chocolates, caramelos, pies, queques) que comes? |
|  | How often does this caregiver Keep track of the quantity of industrialized snacks (potato chips, munchies, cheese pastries, etc.) that you eat? (2) |
|  | ¿Con qué frecuencia tus padres revisan la cantidad de snacks industrializados (papas fritas, ramitas, doritos) que comes? |
|  | How often does this caregiver keep track of the quantity of fatty foods (Hamburgers, snacks, mayonnaise, etc.) that you eat? (3) |
|  | ¿Con qué frecuencia tus padres revisan la cantidad de comida alta en grasa (hamburguesas, snacks, mayonesa) que comes? |
|  | How often does s/he keep track of the quantity of sweet drinks (soda/soft drinks, juices) that you drink? (4) |
|  | ¿Con qué frecuencia tus padres revisan la cantidad de bebidas azucaradas que tomas? |
| **Control Adolescente** | This caregiver allows you to eat whatever you wants? (5) |
|  | ¿Tus padres te permiten comer lo que quieras? |
|  | Can you choose the items you want of what is served at lunch or dinner, leaving aside what you donor like, without interference from the caregiver? (6) |
|  | ¿Puedes elegir los alimentos que quieres comer de lo que se te sirve al almuerzo o cena, dejando a un lado lo que no te gusta? |
|  | When you do not like what is served for eating, does your caregiver cook something else for you? (10) |
|  | ¿Cuando no te gusta lo que se sirve para comer, ¿tus padres preparan algo más? |
|  | Does this caregiver allow you to have snacks whenever you want? (11) |
|  | ¿Tus padres permiten que comas snacks cuando tú quieres? |
| **Restricción para el control de peso** | This caregiver needs to be sure that I do not eat fatty foods. (18) |
|  | ¿Mis padres necesitan estar seguros de que no como alimentos altos en grasa? |
|  | This person encourages me to eat less food so that I won’t get fat. (24) |
|  | Mis padres me animan a que coma menos comida para que no engorde. |
|  | This person helps me controlling the quantity of food that I serve myself at each meal in order to control my weight. (26) |
|  | Mis padres me ayudan a controlar la cantidad de alimentos que me sirvo en cada comida para controlar mi peso. |
|  | If I eat more than normally at one meal, this person reduces the quantity of food at the next meal. (29) |
|  | Si como más de lo normal en una comida, mis padres reducen la cantidad de alimentos de la siguiente comida. |
|  | This caregiver limits the foods that might make me fat. (30) |
|  | Mis padres restringen la comida que podría hacerme engordar. |
|  | S/he believes that I should not eat certain foods so that I do not gain weight. (31) |
|  | Mis padres creen que no debo comer cierta comida porque me hará engordar. |
|  | I am monitored so that I do not eat between meals in order to not get fat. (36) |
|  | Mis padres no me permiten comer entre comidas para no engordar. |
|  | This caregiver -forces me to restrict my diet in order to control my weight. (39) |
|  | Mis padres me ponen a dieta para controlar mi peso. |
| **Modelo Parental** | This caregiver eats healthy food to give me an example of healthy eating habits. (38) |
|  | Mis padres comen comida saludable para darme un ejemplo de alimentación saludable. |
|  | Even when it is not the caregiver's preferred food, s/he often eats it because s/he finds it important to give me her/his example. (40) |
|  | Incluso cuando no es la comida preferida de mis padres, igual se la comen, porque consideran que es importante. |
|  | S/he tries to show enthusiasm regarding healthy food. (41) |
|  | Mis padres tratan de mostrar entusiasmo respecto a la comida saludable. |
|  | S/he shows me how much s/he enjoys eating healthy food. (42) |
|  | Mis padres me muestran cuanto les gusta comer alimentos saludables. |
