# Supplementary material for: Direct and indirect relationships between Food Parental Practices, diet quality, and food satisfaction in adolescents
Source: Front Public Health. 2025 Jan 30;12:1504642. doi: 10.3389/fpubh.2024.1504642 (PMC11822477; doi:10.3389/fpubh.2024.1504642)
Supplement: Supplementary file 2 [file Table_2.doc]

Supplementary Material

# Supplementary Tables

**Adapted Healthy Eating Index (AHEI) (Kennedy et al., 1995), adapted by (Norte & Ortíz. 2011)**

| **Variables** | **Puntajes** | **Recomendación** |
| --- | --- | --- |
| Cereales y derivados | 0 = “Nunca o casi nunca”  2,5 = “Menos de una vez por semana”  5 = “Una o dos veces por semana”  7,5 = “Tres veces por semana”  10 = “Todos los días” | Consumo diario |
| Verduras y hortalizas | 0 = “Nunca o casi nunca”  2,5 = “Menos de una vez por semana”  5 = “Una o dos veces por semana”  7,5 = “Tres veces por semana”  10 = “Todos los días” | Consumo diario |
| Frutas | 0 = “Nunca o casi nunca”  2,5 = “Menos de una vez por semana”  5 = “Una o dos veces por semana”  7,5 = “Tres veces por semana”  10 = “Todos los días” | Consumo diario |
| Leche y derivados | 0 = “Nunca o casi nunca”  2,5 = “Menos de una vez por semana”  5 = “Una o dos veces por semana”  7,5 = “Tres veces por semana”  10 = “Todos los días” | Consumo diario |
| Carne | 0 = “Nunca o casi nunca”  2,5 = “Todos los días”  5 = “Menos de una vez por semana”  7,5 = “Tres veces por semana”  10 = “Una o dos veces por semana” | Consumo semanal |
| Legumbres (ej: porotos, lentejas, arvejas) | 0 = “Nunca o casi nunca”  2,5 = “Todos los días”  5 = “Menos de una vez por semana”  7,5 = “Tres veces por semana”  10 = “Una o dos veces por semana” | Consumo semanal |
| Embutidos y fiambres (ej.: jamón, mortadela, longanizas, vienesas, paté) | 0 = “Todos los días”  2,5 = “Tres veces por semana”  5 = “Una o dos veces por semana”  7,5 = “Menos de una vez por semana”  10 = “Nunca o casi nunca” | Consumo Ocasional |
| Dulces | 0 = “Todos los días”  2,5 = “Tres veces por semana”  5 = “Una o dos veces por semana”  7,5 = “Menos de una vez por semana”  10 = “Nunca o casi nunca” | Consumo Ocasional |
| Refrescos con azúcar | 0 = “Todos los días”  2,5 = “Tres veces por semana”  5 = “Una o dos veces por semana”  7,5 = “Menos de una vez por semana”  10 = “Nunca o casi nunca” | Consumo Ocasional |
| Variedad de la dieta | 2 = Cumple con c/u de las recomendaciones diarias  1 = Cumple con c/u de las recomendaciones semanales |  |
