# Supplementary material for: Direct and indirect relationships between Food Parental Practices, diet quality, and food satisfaction in adolescents
Source: Front Public Health. 2025 Jan 30;12:1504642. doi: 10.3389/fpubh.2024.1504642 (PMC11822477; doi:10.3389/fpubh.2024.1504642)
Supplement: Supplementary file 3 [file Table_3.doc]

Supplementary Material

# Supplementary Tables

**Satisfaction With Food related-Life (SWFoL) adapted and validate by** Schnettler et al. (2013)

| **Ítems** | **SWFoL** | **Likert** |
| --- | --- | --- |
| 1 | Los alimentos y comidas son elementos muy positivos en tu vida | 1 - 6 |
| 2 | Estás muy complacido/a con tu alimentación | 1 - 6 |
| 3 | Tu vida en relación a los alimentos y comidas se acerca al ideal | 1 - 6 |
| 4 | Respecto a los alimentos, tu condición de vida es excelente | 1 - 6 |
| 5 | Los alimentos y comidas te proporcionan gran satisfacción en tu vida diaria | 1 - 6 |
